# Anti-pathogenic potential of a classical *ayurvedic* *Triphala *formulation

**DOI:** 10.12688/f1000research.19787.2

**Published:** 2020-10-01

**Authors:** Hinal Patel, Foram Patel, Vinit Jani, Neha Jha, Afsa Ansari, Bhumika Paliwal, Bharatsingh Rathod, Dhruvi Patel, Pooja Patel, Vijay Kothari

**Affiliations:** 1 Institute of Science, Nirma University, Ahmedabad, Gujarat, 382481, India

**Keywords:** Antimicrobial resistance (AMR), Quorum Sensing (QS), Triphala, Polyherbal, Post Extract Effect (PEE), Anti-virulence, Lysozyme

## Abstract

A classical *ayurvedic* polyherbal formulation namely *Triphala* was assessed for its anti-pathogenic potential against five different pathogenic bacteria. Virulence of four of them towards the model host *Caenorhabditis elegans* was attenuated (by 18-45%) owing to pre-treatment with *Triphala* Formulation (TF) (≤20 µg/ml). TF **could also exert significant therapeutic effect on worms already infected with *Chromobacterium violaceum *(MTCC 2656), *Serratia marcescens* (MTCC 97) or *Staphylococcus aureus *(MTCC 737). Prophylactic use of TF **allowed worms to score 14-41% better survival in face of subsequent pathogen challenge. Repeated exposure to this formulation induced resistance in *S. marcescens*, but not in *P. aeruginosa*. It also exerted a post-extract effect (PEE) on three of the test pathogens. TF was able to modulate production of quorum sensing (QS)-regulated pigments in three of the multidrug-resistant gram-negative test bacteria. Haemolytic activity of *S. aureus* was heavily inhibited under the influence of this formulation. *P. aeruginosa's* lysozyme-susceptibility was found to increase by ~25-43% upon TF-pretreatment. These results validate therapeutic potential of one of the most widely used polyherbal *ayurvedic *formulations called *Triphala*.

## Background

Antibiotic-resistant bacterial infections are among the most serious public-health threats. Since the emergence and spread of antimicrobial resistance (AMR) is shrinking the utility spectrum of conventional bactericidal antibiotics, there is an urgent need for discovery and development of novel anti-virulence formulations. Traditional Medicine (TM) systems like *Ayurveda* offer several sophisticated formulations for a variety of disease conditions. One such classical ayurvedic formulation with a long history of safe use is *Triphala*. *Triphala* is a polyherbal formulation containing three myrobalans fruits i.e. *Phyllanthus emblica*, *Terminalia bellerica* and *Terminalia chebula* ( Patwardhan *et al*., 2015). TF is prescribed as a general health promoter, for management of metabolic disorders, dental and skin problems, and for wound management. It has been reported to be active against bacterial pathogens of urinary tract ( Bag *et al*., 2013), and as an anticaries agent for control of gum infections ( Bhattacharjee *et al*., 2015; Prakash & Shelke, 2014). Though many popular formulations like *Triphala* have been used historically in TM and as a household remedy, their validation through modern scientific methods is necessary for their wider acceptance in modern medicine ( Kothari, 2018). This study aimed to investigate the anti-pathogenic efficacy of TF against five different pathogenic bacteria.

## Methods

### Test formulation

*Triphala* formulation (TF) (Emami Ltd; batch no. EM0029; Proportion of 3 constituent plant species: 1:1:1 i.e. *P. emblica*, *T. bellerica*, and *T. chebula*) was purchased from a local market in Ahmedabad, India). For assay purpose, 150 mg of this formulation was suspended in 5 ml of DMSO (Merck, Mumbai), followed by vortexing for 15 min. Then it was centrifuged at 8,000 rpm for 30 min at ambient temperature, and resulting supernatant was collected in a sterile 15 ml glass vial (Borosil) and stored under refrigeration till further use. Remaining pellet was subjected to drying in an oven at 70-80°C until the solvent was completely evaporated, followed by weighing of the dried plant material. Subtracting the latter from the initial weight of 150 mg, the concentration of test formulation in supernatant was calculated to be 22.94 mg/ml. This way the whole formulation was found to contain 70% DMSO soluble fraction, which was used for our experiments.

### Bacterial strains

*a.* Pathogenic bacteria: *Chromobacterium violaceum* (MTCC 2656), *Serratia marcescens* (MTCC 97), *Staphylococcus aureus* (MTCC 737), and *Streptococcus pyogenes* (MTCC 1924) were procured from Microbial Type Culture Collection (MTCC), Chandigarh. *Pseudomonas aeruginosa* was available in our institutional culture collection. All the three gram-negative bacteria used in this study were multidrug resistant, and their antibiogram has previously been reported by us ( Joshi *et al*., 2019; Patel *et al*., 2019a). Additionally, *C. violaceum* and *S. marcescens* strains mentioned here have been reported by us as beta-lactamase producers ( Sarvaiya & Kothari, 2017).
*b.* Probiotic bacteria: *Bifidobacterium bifidum* (NCDC 255), *Enterococcus faecium* (NCIM 5366), and *Lactobacillus plantarum* (MTCC 2621)
NCDC: National Collection of Dairy Cultures, Karnal, India
NCIM: National Collection of Industrial Microorganisms, Pune, India

### *In vivo* assays

*In vivo* efficacy of TF against bacterial infections was tested in the nematode host *Caenorhabditis elegans* N2-Bristol (maintained at the Institute of Science, Nirma University). Maintenance and synchronization of the worm population was done as previously described in Joshi (2019). Worms were maintained on NGM [Nematode Growing Medium; 2.5 g/L peptone (HiMedia, RM001-500G), 3 g/L NaCl (HiMedia, MB023-500G), 1 M CaCl _2 _(HiMedia, GRM534-500G), 1 M MgSO _4 _(Merck, 1.93645.0521), 5 mg/mL cholesterol (HiMedia, TC101-5G), 1 M phosphate buffer of pH 6, 17 g/L agar-agar (HiMedia, GRM666-500G)] agar plate with *E. coli* OP50 (LabTIE B.V. OP50 V.2; batch # 002, JR Rosmalen, the Netherlands) as food. For synchronization of the worm population, adult worms from a 4–5 days old NGM plate were first washed with distilled water, and then treated with 1 mL of bleaching solution [1N NaOH (HiMedia MB095-100G) + 4% NaOCl (Merck 61842010001730) + water in 1:1:3 proportion], followed by centrifugation (at 1500 rpm at 22°C) for 1 min. Eggs in the resultant pellet were washed with sterile distilled water, and then transferred onto a new NGM plate seeded with *E. coli* OP50. L3-L4 stage worms appearing on this plate after 2–3 days of incubation at 22°C were used for further experiments.

Three different types of *in vivo* assays were done as under, employing the methodology described in reference cited in parenthesis following the assay name:

a. Anti-infective assay ( Patel *et al*., 2018a): TF exposed-pathogenic bacteria were allowed to infect *C. elegans* (L3-L4 stage), and their capacity to kill the worm population was compared with their TF-unexposed counterparts, over a period of 5 days.
b. TF as a post-infection therapy ( Patel *et al*., 2019b): Worms already infected with pathogenic bacteria not previously exposed to the test formulation were treated with TF to see whether the test formulation can exert any therapeutic effect on already infected worms. Assay methods remained the same as described in previous section, except that TF was added into assay wells after allowing bacteria either for 6 h or 24 h to establish infection.
c. Prophylactic assay ( Patel *et al*., 2018b): TF-fed worms were challenged with pathogenic bacteria previously not exposed to the test formulation, and their ability to survive in face of pathogen challenge was compared with their TF-unfed counterparts. *C. elegans* worms maintained on NGM were kept unfed for 24 h prior to being used for experiments. These worms were then fed with TF by mixing required concentration of this formulation (100 µL of DMSO-dissolved *Triphala*) with 800 µL of M9 medium and placed in a sterile non-treated polystyrene 24-well plate (HiMediaTPG24-1X50NO) containing 10 worms per well. Duration of exposure of worms to TF was kept to 96 h, followed by addition of 100 µL of pathogenic bacterial suspension of OD _764_= 1.50 measured with Agilent Cary 60 UV-Vis spectrophotometer). Appropriate controls i.e. worms previously not exposed to TF *,* but exposed to pathogenic bacteria; worms exposed neither to TF nor bacteria; and worms exposed to TF, but not to bacterial pathogens, were also included in the experiment. Incubation was carried out at 22°C. Number of dead vs. live worms were counted every day for 5 days by putting the plate with lid under a light microscope 4X objective (Catalyst Biotech CatScope CS-U207T). Straight worms were considered to be dead. Plates were gently tapped to confirm lack of movement in the apparently-dead worms. On the last day of the experiment, when plates could be opened, their death was also confirmed by touching them with a straight wire, wherein no movement was taken as confirmation of death.

Catechin (Sigma Aldrich; C1251-5G) and standard antibiotics (HiMedia; Ampicillin CMS645-1G; Gentamicin TC026-1G; Chloramphenicol CMS218-5G; Vancomycin CMS217-500MG) were used as positive controls. Catechin was employed at 100 µg/ml, whereas different sub-MIC concentrations (0.1–5 µg/ml) of the antibiotics were used against different organisms as per their susceptibility.

Videos of some of the *in vivo* assays were captured on the fifth day of the experiment, using an inverted microscope (Nikon Eclipse Ti) under 4X objective lens, wherein 100 µl of the liquid content from 24-well assay plate was transferred onto a large cover slip for observation and video capturing [see extended data ( Patel *et al*., 2019c)].

### *In vitro* assays

After confirming the *in vivo* anti-pathogenic efficacy of the TF, we performed following *in vitro* assays to gain insight into interaction of this formulation with the pathogenic bacteria, as per the methodology described in respective references mentioned in the parenthesis:

a. Broth dilution assay ( Joshi *et al*., 2016) to investigate effect of TF on bacterial growth and quorum sensing (QS)-regulated pigment production: *C. violaceum*, and *S. marcescens* were inoculated in nutrient broth (HiMedia MV002-500G) supplemented with TF. Media used for *P. aeruginosa* and *S. aureus* were Pseudomonas broth [10 g/l potassium sulfate (SRL 44277), 1.4 g/l magnesium chloride (Merck 1.9366.30521), 16 g/l peptone (HiMedia RM001-500G)], and Staphylococcus broth (HiMedia M578-500G) respectively. *S. pyogenes* was grown in BHI (brain heart infusion; HiMedia MV210-500G) broth. Following incubation, cell density was measured at 750 nm in a microplate reader (Biorad 680). Pigment from these culture broths were extracted as previously described by us in Joshi *et al.* (2016). Cell pellets of *C. violaceum*, *S. marcescens*, or *S. aureus* were dissolved in appropriate solvent i.e. *C. violaceum* pellet in DMSO [(Merck 1.07046.2521); *S. marcescens* pellet in acidified methanol [4 ml HCl (HiMedia AS003-500ML) into 96 ml of methanol]; and *S. aureus* pellet in methanol (Merck 1.94516.2521). This allowed their pigments to be extracted in the solvent applied. In case of *P. aeruginosa*, pigment extraction was achieved by mixing chloroform (Merck 1.67024.0521) with culture broth (2:1 ratio). Quantification of each pigment was done at the wavelength nearest to its λ _max, _available in the microplate reader (Biorad 680) used by us.
b. Effect of TF on biofilm formation and its possible potential to eradicate pre-formed biofilm was assessed through crystal violet assay ( Patel *et al*., 2013); and its effect on biofilm viability was assessed through MTT assay ( Trafny *et al*., 2013). For the crystal violet assay, the biofilm-containing tubes after discarding the inside liquid were washed with PBS in order to remove all nonadherent bacteria and air-dried for 15 min. Then, each of the washed tubes was stained with 1.5 mL of 0.4% aqueous crystal violet solution (SRL 54862-9) for 30 min. Afterwards, each tube was washed twice with 2 ml of sterile distilled water and immediately destained with 1500 μL of 95% ethanol. After 45 min of destaining, 1 mL of destaining solution was transferred into separate tubes and read at 570 nm in a microplate reader (Biorad 680). For the MTT assay, the biofilm-containing tubes (after discarding the inside liquid) were washed with PBS in order to remove all nonadherent bacteria and air-dried for 15 min. Then, 900 µL of minimal media was added into each tube, followed by addition of 100 μL of 0.3% MTT (3-(4,5-dimethylthiazol-2-yl)-2,5-diphenyltetrazolium bromide; HiMedia MB186-100MG). After 2 h incubation at 37°C, resulting purple formazan derivatives were dissolved in DMSO and measured at 570 nm in a microplate reader (Biorad 680).
c. Effect of TF on haemolytic potential of the test pathogens ( Neun *et al*., 2015): Small volume of human blood required for this assay was obtained from the authors, who each gave their written informed consent. The use of this blood was approved by the Institutional Ethics Committee of the Institute of Science, Nirma University (approval no: EC/NU/18/IS/4). Blood collection was executed by one of the authors (AA) on three different times in heparinized vials. OD _750_ of the overnight culture grown in presence or absence of TF was standardized to 1.00. Cell-free supernatant was prepared by centrifugation at 15,300 g for 10 min. 10 μl of human blood was incubated with this cell-free supernatant for 2 h at 37°C, followed by centrifugation at 800 g for 15 min. OD of the supernatant was read at 490 nm in a microplate reader (Biorad 680), to quantify the amount of hemoglobin released. 1% Triton X-100 (CDH, New Delhi; CDH030632) was used as a positive control. Phosphate buffer saline was used as a negative control.
d. Effect of TF on lysozyme-susceptibility of test pathogens: Bacterial cells were first inoculated in a TF-containing media for 24 h, and then the cell pellet was separated by centrifugation [at 15,000 rpm for 15 min] to be resuspended into phosphate buffer saline (PBS) of pH 7.4, so as to attain OD _750_=1 (Biorad 680). 200 µl of this bacterial suspension was mixed with 750 µg/ml lysozyme (Sigma Aldrich L6876-1G) prepared in PBS, and then incubated for 24 h at appropriate temp for each organism. At the end of incubation OD _750 _was measured.

### Effect of TF on probiotic strains

*Bifidobacterium bifidum* (NCDC 255), *Enterococcus faecium* (NCIM 5366), and *Lactobacillus plantarum* (MTCC 2621) were grown in Lactobacillus MRS broth (HiMedia GM369-500G) containing TF, in screw capped tubes at 37°C for 22-24 h. For *B. bifidum*, this medium was supplemented with 0.05% cysteine (HiMedia PCT0305-25G). Effect of TF (10–100 µg/ml) on these bacteria was interpreted by comparing their cell density [OD _655 _measured with microplate reader (Biorad 680)] in TF-supplemented media to that in TF-free media.

### Statistical analysis

All the experiments were performed in triplicate, and measurements are reported as mean ± standard deviation (SD) of 3 independent experiments. Statistical significance of the data was evaluated by applying *t*-test using Microsoft Excel ^® ^2013. *p* values ≤0.05 were considered to be statistically significant. Trial version of GraphPad Prism 7 was used to make Kaplan-Meier survival curve for worms.

## Results

### *In vivo* experiments

**Figure 1.**
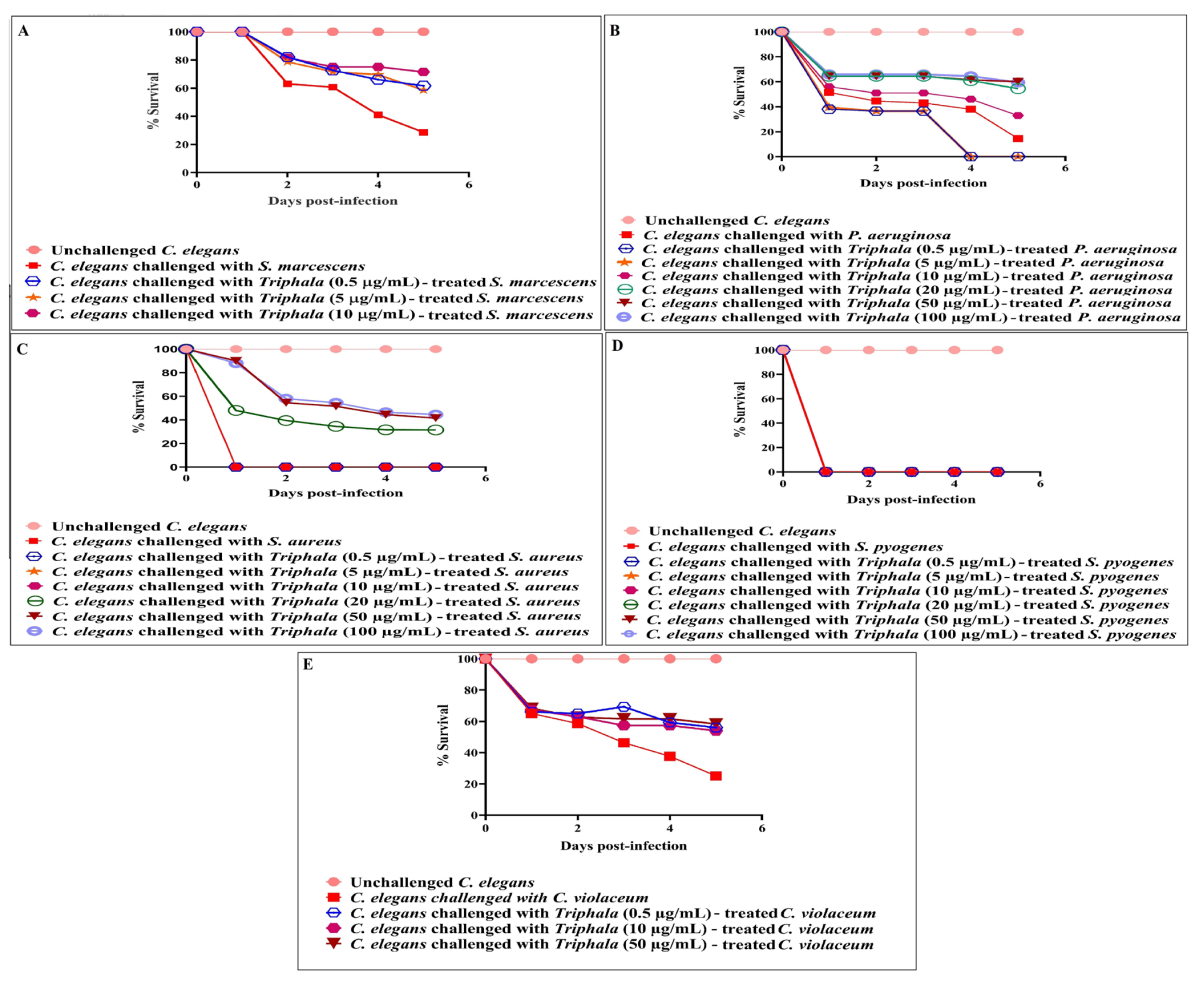
Anti-infective activity of *Triphala* formulation against test pathogens. TF-treatment attenuated virulence of four of the test pathogens towards *C. elegans*. DMSO present in the ‘vehicle control’ at 0.5%v/v did not affect virulence of any of the bacterium towards *C. elegans*; DMSO (0.5%v/v) and TF at tested concentrations showed no toxicity towards the worm. ( **A**) TF at 0.5 µg/ml, 5 µg/ml, 10 µg/ml, 20 µg/ml, 50 µg/ml, and 100 µg/ml allowed 32%±5.10, 29%***±9.60, 41.9%***±2.40, 38.4%***±7.10, 39.9%***±9.40, and 41.4%***±7.10 better survival of the worm population respectively, when challenged with *S. marcescens.* Also see videos a-b submitted as part of extended data. ( **B**) TF at 10 µg/ml, 20 µg/ml, 50 µg/ml, and 100 µg/ml allowed 18%***±5.01, 40%***±7.01, 45.5%***±0, and 45%***±5.00 better survival of the worm population respectively, when challenged with *P. aeruginosa.* ( **C**) TF at 20 µg/ml, 50 µg/ml and 100 µg/ml allowed 31.5%±***2.35, 41.5%***±2.35and 44.5%***±14.14 better survival of the worm population respectively, when challenged with *S. aureus.* ( **D**) TF-treatment did not attenuate virulence of *S. pyogenes* towards the worms. ( **E**) TF at 0.5µg/ml, 5 µg/ml, 10 µg/ml, 20 µg/ml, 50 µg/ml, and 100 µg/ml allowed 31%***±5.77, 29.5%***±8.81, 29%***±1.92, 29%***±1.92, 32.3%***±1.92, and 32.3%**±5.09 better survival of the worm population respectively, when challenged with *C. violaceum.* *p<0.05, **p<0.01, ***p<0.001; TF: *Triphala* Formulation.

**Figure 2.**
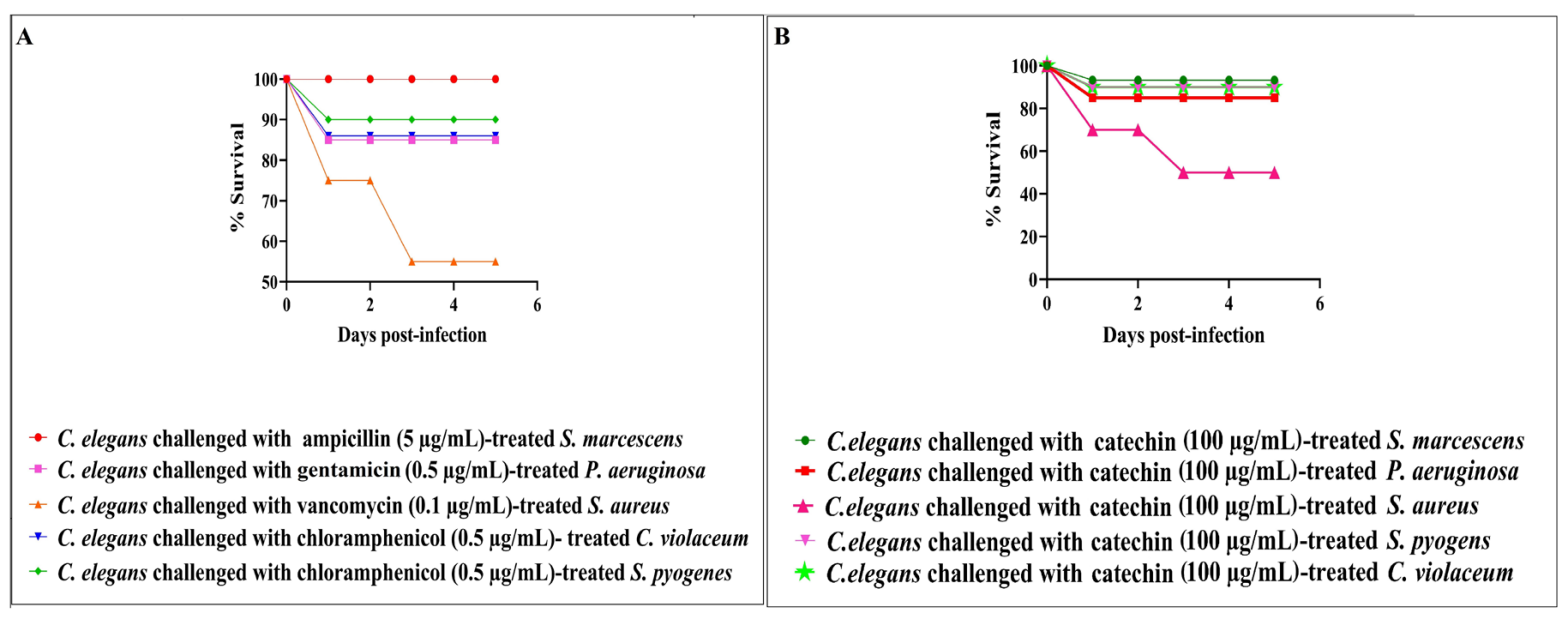
Anti-infective activity of positive controls (catechin and antibiotics) against test pathogens. Catechin was able to reduce virulence of different test bacteria towards *C. elegans* by 50–100% (p≤0.05). Various standard antibiotics at sub-MIC level could reduce bacterial virulence by 55–100% (p≤0.05).

***Anti-infective assay.*** When all the five pathogens were pre-treated with 0.5-100 µg/ml of TF before being allowed to attack *C. elegans, Triphala* formulation (TF) was able to attenuate virulence of all test pathogens except *S. pyogenes* at ≤20 µg/ml [ Figure 1; underlying data ( Patel *et al*., 2019c)]. Worms challenged with TF-treated pathogens demonstrated 18–45.50% better survival than those challenged with TF-unexposed pathogens. Effect of catechin and standard antibiotics used as positive controls on bacterial virulence is shown in Figure 2 [underlying data ( Patel *et al*., 2019c)].

**Figure 3.**
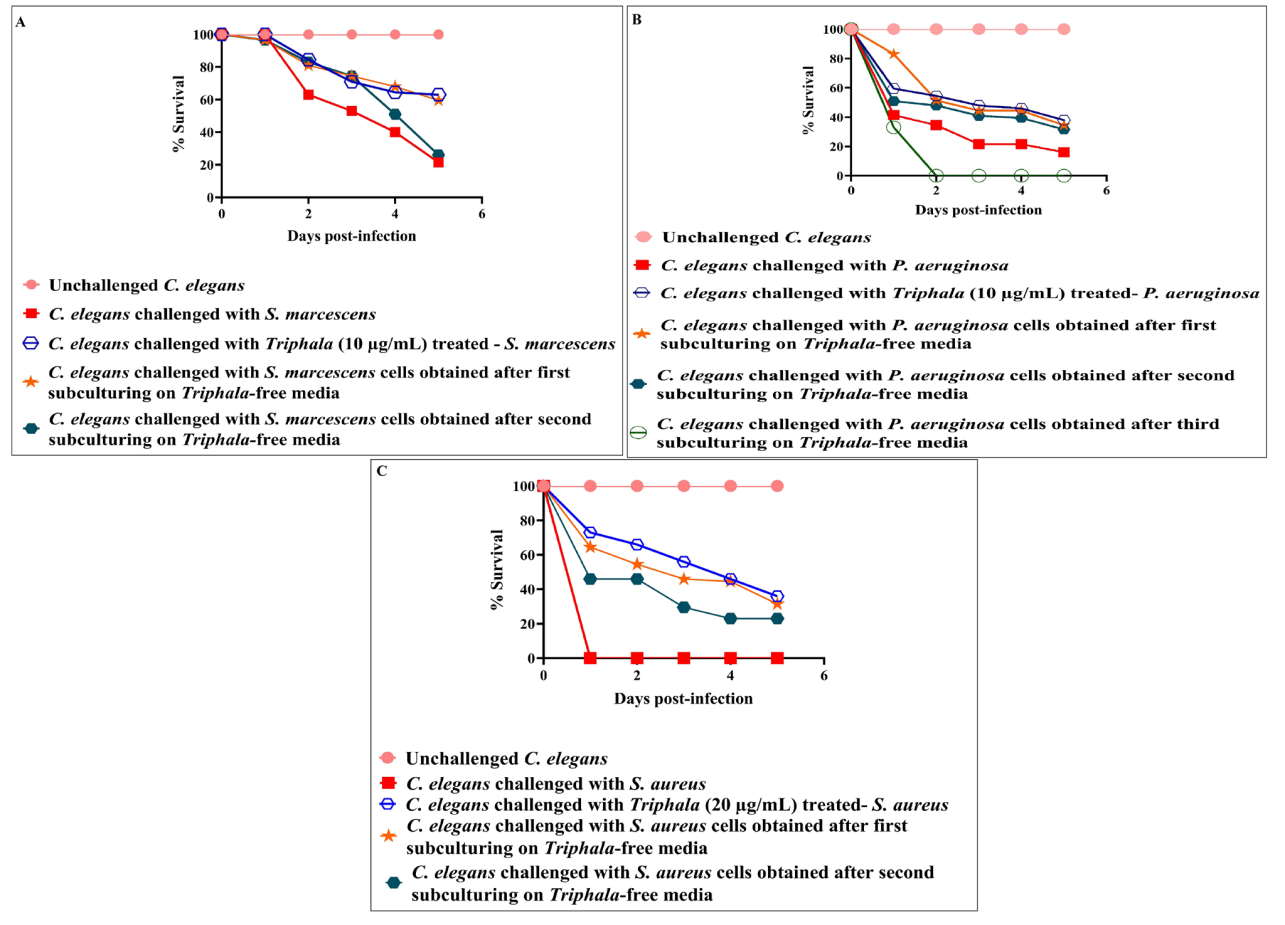
Post extract effect of *Triphala* on test pathogens. TF-treatment reduces the virulence of all the test pathogens towards *C. elegans* even after subculturing of cells in TF-free media. DMSO present in the ‘vehicle control’ at 0.5% v/v did not affect virulence of the bacteria towards *C. elegans*; DMSO (0.5%v/v) and TF at tested concentrations showed no toxicity towards the worm. ( **A**) *S. marcescens* obtained after first subculturing on TF-free media were able to kill 38%***±4.71 lesser worms than control; ( **B**) *P. aeruginosa* obtained after first and second subculturing on TF-free media were able to kill 18.5%**±2.35 and 15.5%*±2.35 lesser worms respectively, than control; ( **C**) *S. aureus* obtained after first and second subculturing on TF-free media were able to kill 31.5%***±2.35 and 23%***±0 lesser worms respectively, than control. *p<0.05, **p<0.01, ***p<0.001; TF: *Triphala* Formulation.

 After confirming the anti-infective activity of TF, we investigated (as described in Patel *et al*., 2019a) whether this formulation exerts any post-extract effect (PEE - https://doi.org/10.32388/359873) on the test pathogens i.e. whether the virulence-attenuating effect suffered by the parent bacterial culture is retained even in their daughter population never receiving any direct exposure to TF. When the TF-treated bacteria were subsequently subcultured on TF-free media, their daughter populations were unable to exert virulence at par with that of control (DMSO-treated parent culture having no TF-exposure). In case of *P. aeruginosa* and *S. aureus*, this PEE lasted up to the second subculturing, whereas in the case of *S. marcescens* PEE lasted until first subculturing [ Figure 3; underlying data ( Patel *et al*., 2019c)].

**Figure 4.**
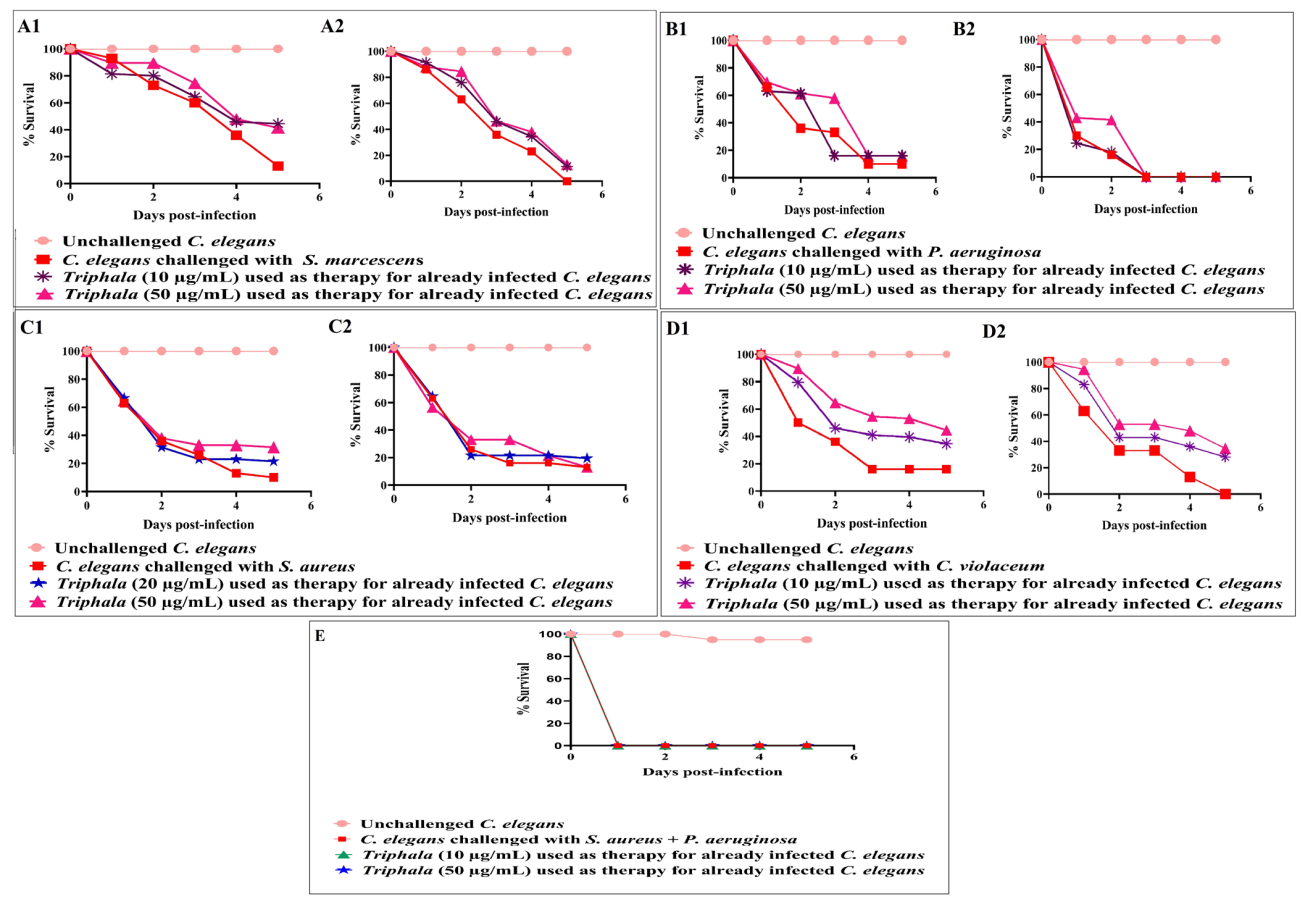
Assessing efficacy of *Triphala* formulation as a post-infection therapy in pre-infected worms. DMSO (0.5%v/v) did not affect survival of pre-infected worms. DMSO (0.5%v/v) and TF at tested concentrations showed no toxicity towards the worm. **A1**– **D1**: TF employed 6 h post-infection **A2**– **D2**: TF employed 24 h post-infection ( **A1**) TF at 10 µg/ml, and 50 µg/ml used as therapy for already *S. marcescens* infected *C. elegans* after 6 h incubation conferred 31.5%***±2.35 and 28.5%***±2.35 survival benefit, respectively; ( **A2**) TF at 10 µg/ml, and 50 µg/ml used as therapy for already *S. marcescens* infected *C. elegans* after 24 h incubation conferred 11.5%***±2.35 and 13%*** ±0.51 survival benefit, respectively; ( **B1**– **B2**):TF could not rescue *P. aeruginosa*-infected worms when tested as post-infection remedy. ( **C1**) TF at 20 µg/ml, and 50 µg/ml used as therapy for already *S. aureus*-infected *C. elegans* after 6 h incubation conferred 11.5%***±2.35 and 21.5%***±2.35 survival benefit, respectively; ( **C2**) TF could not rescue *S. aureus* infected worms when tested as post-infection remedy. ( **D1**) TF at 10 µg/ml, and 50 µg/ml used as therapy for already *C. violaceum* infected *C. elegans* after 6 h incubation conferred 18.5%***±2.35 and 28.5%***±2.35 survival benefit, respectively; ( **D2**) TF at 10 µg/ml, 50 µg/ml used as therapy for already *C. violaceum* infected *C. elegans* after 24 h incubation conferred 22%***±2.35 and 28.5%***±2.35 survival benefit, respectively. ( **E**) TF could not rescue the worms in face of mix-culture infection by *S. aureus* and *P. aeruginosa*, when tested as post-infection remedy. Survival benefit refers to the difference between number of worms surviving in experimental and control wells. *p<0.05, **p<0.01, ***p<0.001; TF: *Triphala* Formulation.

***TF as a post-infection therapy.*** To test the therapeutic efficacy of TF in already-infected worm population, we first allowed different pathogenic bacteria, not previously exposed to TF, to infect *C. elegans* either for 6 h or 24 h, and then exposed the infected worms to two different concentrations of TF. TF failed to exert any therapeutic effect on worms infected with *P. aeruginosa*. However, it could exert significant (p≤0.05) therapeutic effect on worms infected with *C. violaceum* or *S. marcescens*. Against *S. aureus*, TF was effective only if the worms were given TF-treatment early (i.e. 6 hour-post infection) [ Figure 4; underlying data ( Patel *et al*., 2019c)]. TF could also not rescue the worms if they already had a mixed infection ( *S. aureus* and *P. aeruginosa*).

**Figure 5.**
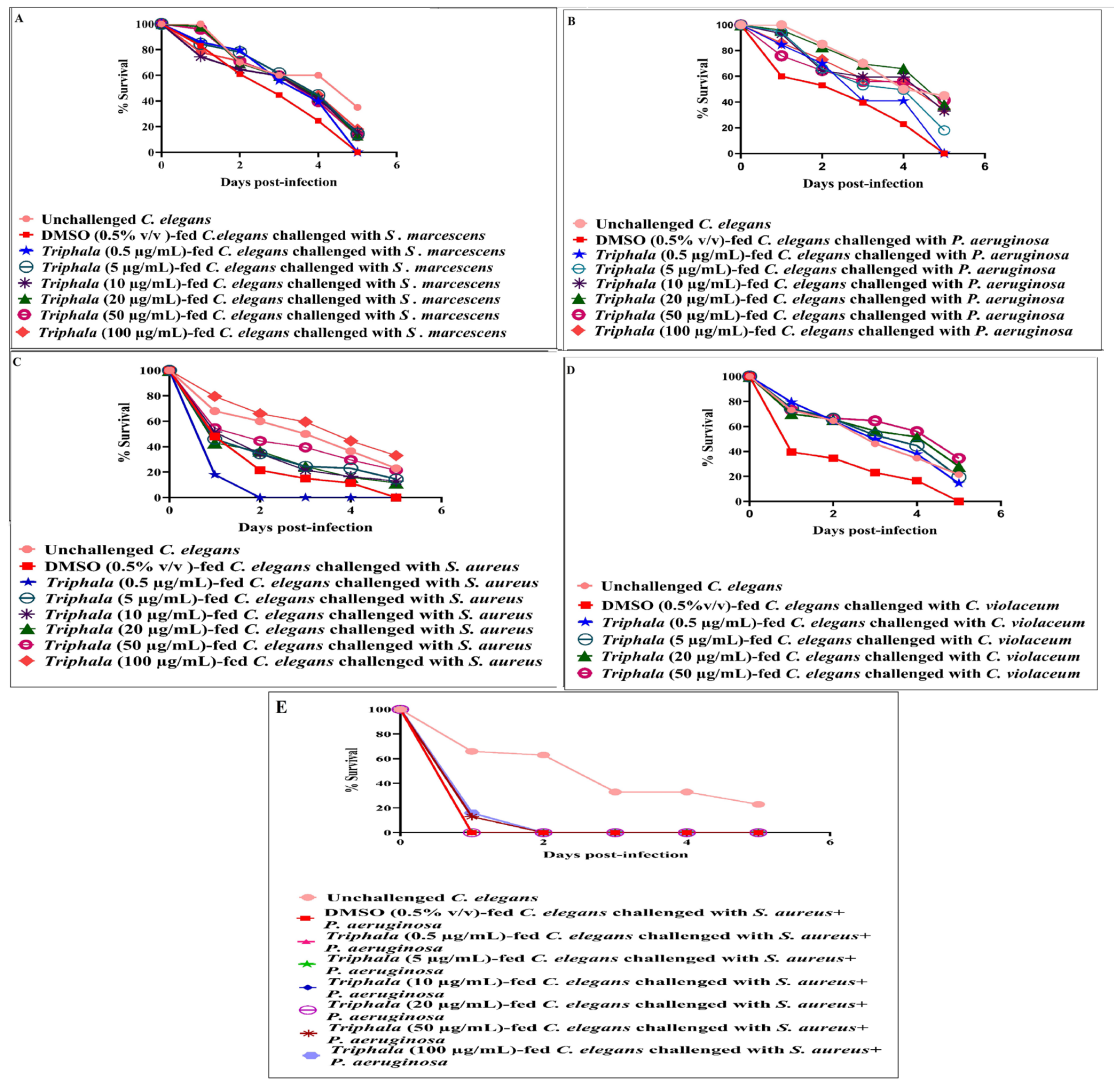
TF-pre-exposed *C. elegans* exhibit better resistance to subsequent bacterial challenge. Pre-treatment of worms with DMSO (0.5%v/v) did not alter their susceptibility to subsequent challenge with pathogenic bacteria. DMSO (0.5%v/v) and TF at tested concentrations showed no toxicity towards the worm. Among positive controls, catechin (100 μg/ml) pre-treatment conferred 23%±0 protection on worm population against subsequent *S. marcescens, P. aeruginosa, S. aureus,* and *C. violaceum* challenge; ( **A**) Ampicillin (5 μg/ml) pre-treatment conferred 26%±0 protection on worm population against subsequent *S. marcescens* challenge; TF (100 µg/ml) pre-treatment conferred 18%***±2.35 protection on fifth day worm population against subsequent *S. marcescens* challenge; ( **B**) Gentamicin (0.5 μg/ml) pre-treatment conferred 26% protection on worm population against subsequent *P. aeruginosa* challenge; TF pretreatment at 5 µg/ml, 10 µg/ml, 20 µg/ml, 50 µg/ml, and 100 µg/ml, conferred 18%***±0, 33%***±2.35, 38%***±2.35, 41.5%**±0 and 34.5%***±2.35 protection on worm population against subsequent *P. aeruginosa* challenge ( **C**) Vancomycin (0.1 μg/ml) pre-treatment conferred 26% protection on worm population, TF pre-treatment at 5 µg/ml, 10 µg/ml, 20 µg/ml, 50 µg/ml, and 100 µg/ml, conferred 14.5%***±2.35, 13%***±4.71, 11.5%***±2.35, 21.5%***±2.35, and 33%***±0 protection on worm population against subsequent *S. aureus* challenge ( **D**) Chloramphenicol (0.5 μg/ml) pre-treatment conferred 26% protection on worm population against *C. violaceum*; TF pre-treatment at 0.5 µg/ml, 5 µg/ml, 10 µg/ml, 20 µg/ml, 50 µg/ml, and 100 µg/ml, conferred 14.5%***±0, 19.5%***±4.71, 14.5%***±2.35, 28%***±7.07, 34.5%***± 2.35 and 35.5%***±0 protection on worm population against subsequent *C. violaceum* challenge. *p<0.05, **p<0.01, ***p<0.001; TF: *Triphala* Formulation.

**Figure 6.**
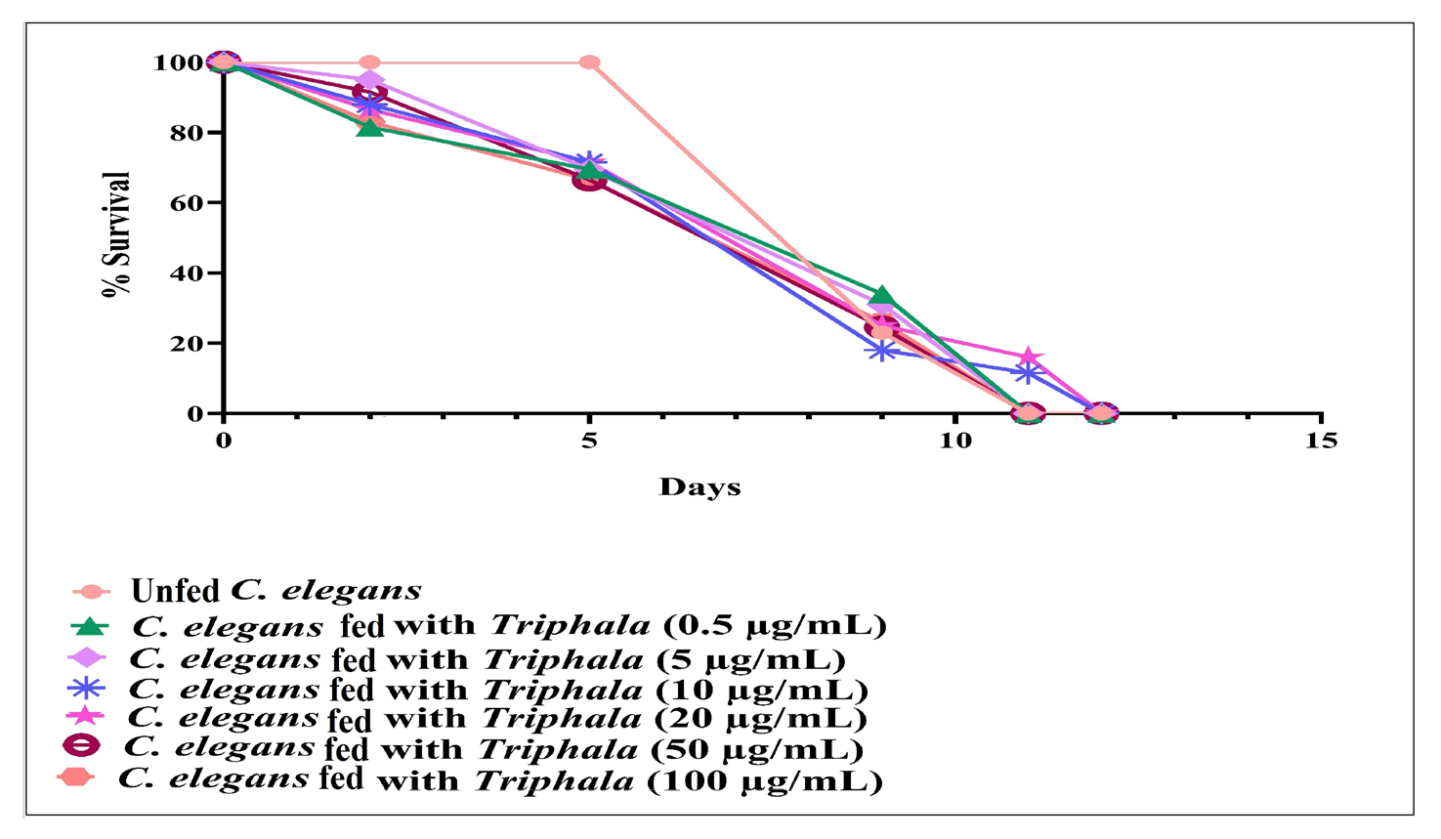
*Triphala* formulation imparts marginal longevity extension on *C. elegans*. Worms fed with TF (10 µg/ml or 20 µg/ml) scored 11.5%***±1.20, and 16%***±0.96 better survival on 11 ^th^ day. All worms (not fed with TF) in control were dead by the 11 ^th^ day. DMSO (0.5%v/v) at tested concentration had no effect on worm longevity. ***p≤0.001; TF: *Triphala* Formulation.

***Prophylactic potential of TF.*** To investigate whether previous feeding with TF can make the worm population tolerate subsequent challenge with pathogenic bacteria, not treated with TF, better; worms were first maintained in a TF-containing M9 buffer for 96 h, and then challenged with different bacterial pathogens. Such TF-fed worms scored 14.50–41.50% better survival in face of pathogen challenge [ Figure 5; underlying data ( Patel *et al*., 2019c)]. However, TF did not confer any prophylactic benefit on the worm population against mix-culture challenge of *P. aeruginosa* and *S. aureus*. Since prophylactic activity of any formulation can be said to stem mainly from its effect on the host, we also compared whether TF imparts any extension of longevity on the worm. Worms fed with TF (10–20 µg/ml) registered marginally better survival up to 11 days ( Figure 6). 

***Repeated exposure of test pathogens to TF.*** Since one of the major challenges with even the most potent antimicrobial agents/formulations is development of resistance against them by the pathogen populations, we tested whether repeated exposure of the test pathogens to TF can induce any resistance in them. For this, we subcultured two of the gram-negative test pathogens ( *P. aeruginosa* and *S. marcescens*) in TF (50 µg/ml)-containing broth for 10 subsequent times, and then the 'TF-habituated' cultures thus obtained were tested for their virulence towards the nematode host. Since antibiotic-resistant strains of *P. aeruginosa* and Entrerobacteriaceae (to which *S. marcences* belongs) are recognized as serious threats ( https://www.cdc.gov/drugresistance/biggest-threats.html; https://www.who.int/medicines/publications/WHO-PPL-Short_Summary_25Feb-ET_NM_WHO.pdf), these two bacteria were used in this assay. Since higher concentration of antimicrobial formulations can be expected to exert higher selection pressure on the susceptible bacterial population, we chose the highest TF concentration (50 µg/ml) beyond which TF does not exert any statistically superior anti-infective effect.

**Figure 7.**
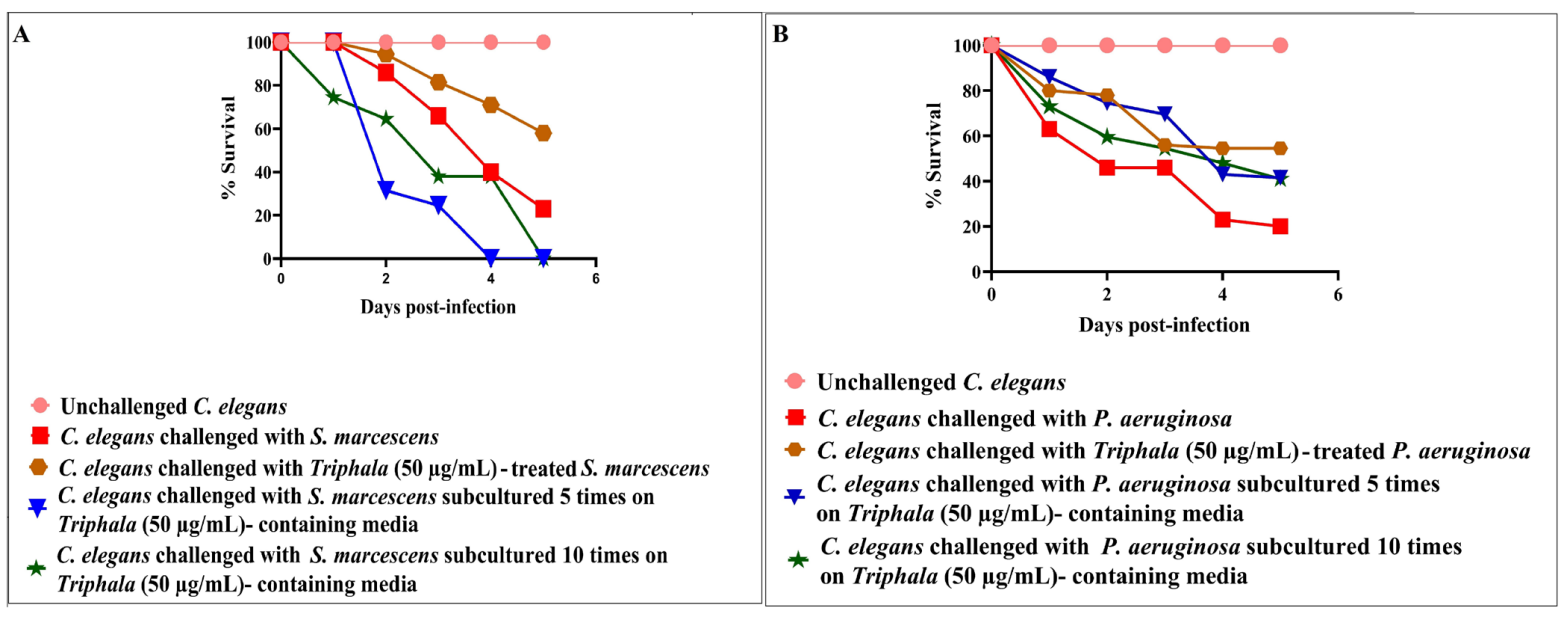
Effect of repeated exposure to *Triphala* formulation on test pathogens. DMSO present in the ‘vehicle control’ at 0.5%v/v did not affect virulence of the bacterium towards *C. elegans*; DMSO (0.5%v/v) and TF at tested concentrations showed no toxicity towards the worms. ( **A**) *C. elegans* challenged with TF (50 μg/ml) treated- *S. marcescens* registered 35%***±2.35 better survival. No survival benefit was available to *C. elegans*, when challenged with *S. marcescens* subcultured 5 times or 10 times on TF (50 μg/ml) containing media. ( **B**) *P. aeruginosa* obtained after 5 and 10 subculturings in TF (50 μg/ml)-containing media were able to kill 21.5%***±2.35 and 21%***±7.07 lesser worms respectively, as compared to control (DMSO-treated) bacterial population. *p<0.05, **p<0.01, ***p<0.001; TF: *Triphala* Formulation.

Repeated TF-exposure was found to induce resistance in *S. marcescens* [ Figure 7A; underlying data ( Patel *et al*., 2019c)]. Though TF-habituated *P. aeruginosa* could kill more worms than its counterpart receiving single TF-exposure, it still could not kill as many worms as TF-unexposed *P. aeruginosa* [ Figure 7B; underlying data ( Patel *et al*., 2019c)]. These results indicate that it may be difficult for the pathogenic bacteria to develop complete resistance against polyherbal formulations like Triphala, but not impossible. These results indicate that it may be difficult for the pathogenic bacteria to develop complete resistance against polyherbal formulations like *Triphala*, but not impossible. Though our previous results on other polyherbal formulations ( Joshi *et al.*, 2019; Patel *et al*., 2019a) and multicomponent crude plant extracts ( Joshi, 2019) have indicated that the probability of pathogens developing resistance against multi-component anti-virulence preparations is low, such a probability can certainly not be ruled out ( Kalia *et al*., 2014; Singh, 2014)

### *In vitro* experiments

**Figure 8.**
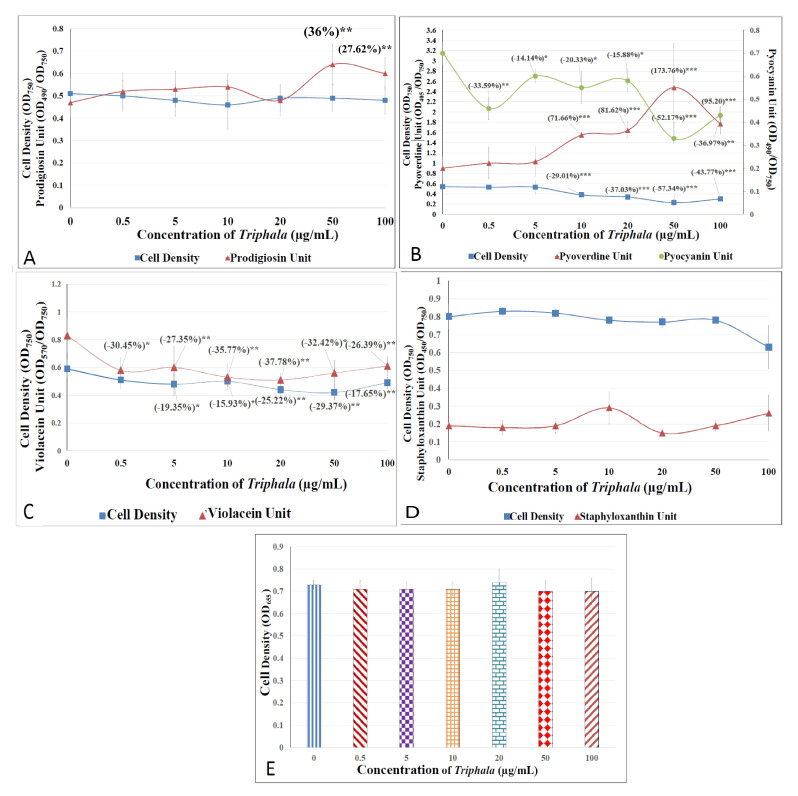
Effect of *Triphala* on growth and QS-regulated pigment production in test pathogens. Bacterial cell density was quantified as OD _750._ DMSO (0.5%v/v) in the vehicle control did not affect growth and pigment production in any of the test pathogens. Pigment Units were calculated to nullify the effect of any change in cell density on pigment production ( **A**) *S. marcescens*: OD of prodigiosin was measured at 490 nm, and Prodigiosin Unit was calculated as the ratio OD _490_/OD _750 _(an indication of prodigiosin production per unit of growth). Catechin (100 µg/ml) inhibited prodigiosin production by 13.05%*±0.10 without affecting bacterial growth; Ampicillin (5 µg/ml) inhibited growth and prodigiosin production by 8.48%**±0.02 and 40.60%*±0.23, respectively. ( **B**) *P. aeruginosa*: OD of pyoverdine and pyocyanin was measured at 405 nm and at 490 nm. Pyoverdine Unit and Pyocyanin Unit was calculated as the ratio OD _405_/OD _750 _and OD _490_/OD _750 _(an indication of pyoverdine and pyocyanin production per unit of growth). Catechin (100 µg/ml) inhibited, pyoverdine and pyocyanin production by 3.85%*± 0.38 and 12.74%*± 2.60 respectively without affecting bacterial growth; Gentamicin (0.5 µg/ml) inhibited, pyoverdine and pyocyanin production by 10.53%*±2.07 and 57.93%***±6.47 without affecting bacterial growth; ( **C**) *C. violaceum*: OD of violacein was measured at 570 nm, and Violacein Unit was calculated as the ratio OD _570_/OD _750_ (an indication of violacein production per unit of growth). Catechin (100 µg/ml) did not affect growth as well as violacein production; Chloramphenicol (0.5 µg/ml) inhibited growth by 40.31**%±0.44 without affecting violacein production. ( **D**) *S. aureus*: OD of staphyloxanthin was measured at 450 nm, and Staphyloxanthin Unit was calculated as the ratio OD _450_/OD _750_ (an indication of staphyloxanthin production per unit of growth). Catechin (100 µg/ml) and vancomycin (0.1 µg/ml) did not affect growth as well as staphyloxanthin pigment production. ( **E**) *S. pyogenes*: TF or catechin (100 µg/ml) did not affect the growth when measured as OD _655_; Chloramphenicol (0.5 µg/ml) inhibited growth by 7.56%**±3.46. * *p*<0.05, ** *p*<0.01, ***p<0.001; TF: *Triphala* Formulation; QS: Quorum Sensing.

Since TF showed significant *in vivo* anti-pathogenic potential in the *C. elegans* model, we performed various *in vitro* experiments to gain insight into its interaction with the target pathogens. TF was able to modulate production of quorum sensing (QS)-regulated pigments in all the three gram-negative bacteria [ Figure 8; underlying data ( Patel *et al*., 2019c)]. It did so with *S. marcescens* without affecting its growth, which is characteristic of an ideal anti-virulence agent i.e. attenuation of virulence without exerting any selection pressure on the susceptible pathogen. However, TF did exert a growth-inhibitory effect on *P. aeruginosa*, wherein its IC _50 _was observed to be near 50 µg/ml. The quorum modulatory effect of TF on pigment production in *P. aeruginosa* was observed not to be amenable to be described by a linear dose-response curve. It seems to fall within the realm of hormesis ( Calabrese, 2004). For example, production of both pigments was not affected maximally at the highest test concentration. Pyocyanin production was inhibited more at 0.5 µg/ml than at 20 µg/ml. Effect of TF on pyoverdine production followed a linear threshold model within concentration range of 0.5–50 µg/ml, but it took an inverted-U shape over 20-100 µg/ml. Though the exact mechanism responsible for a non-linear dose-response relationship is not known, it may be the varying magnitude of bacterial adaptive response at different concentrations of the test agent, which generates such non-linear response curves ( Lushchak, 2014).

**Figure 9.**
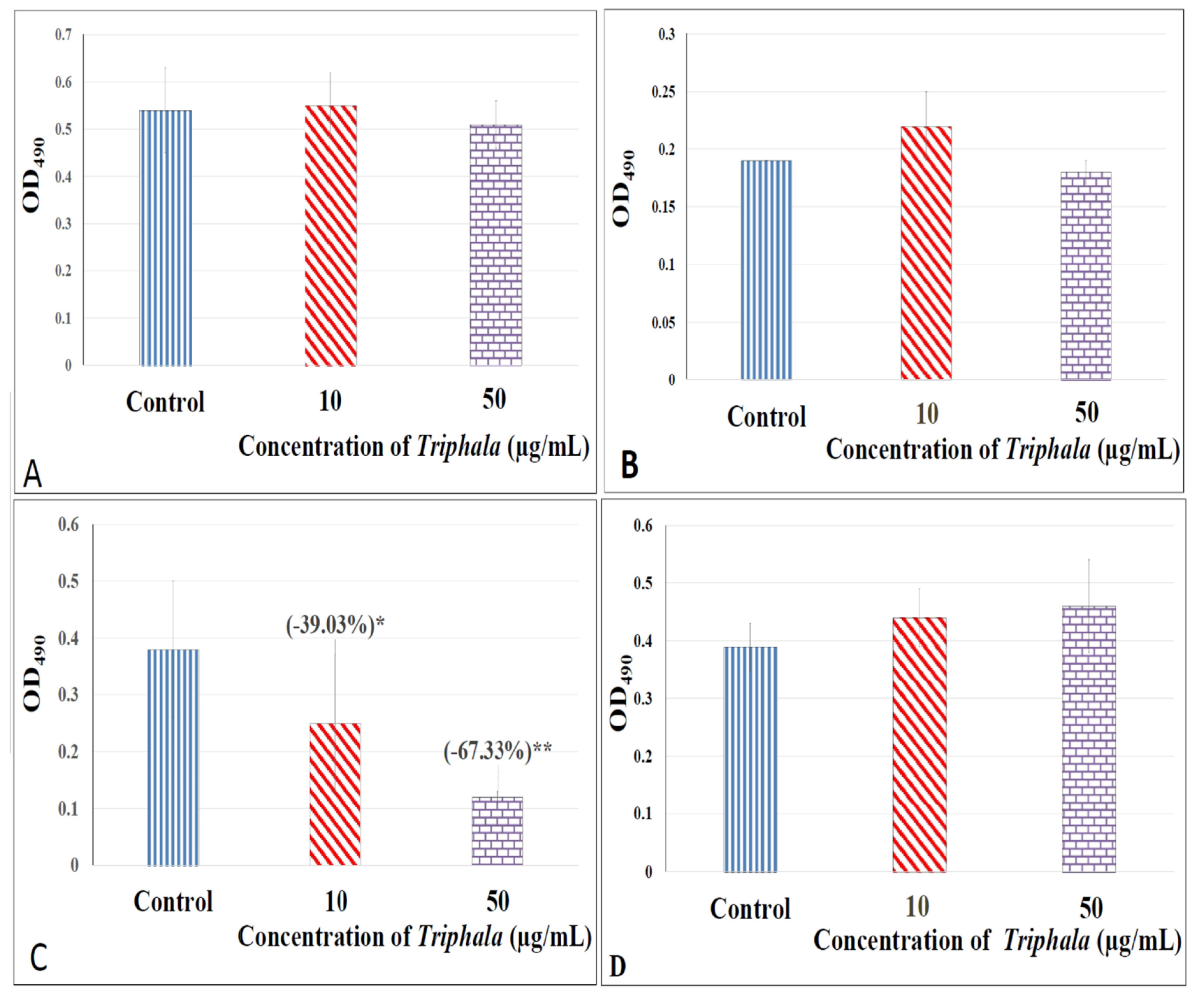
Effect of *Triphala* on haemolytic activity of test pathogens. TF had no effect on hemolytic activity of *S. marcescens* ( **A**), *P. aeruginosa* ( **B**), and *C. violaceum* ( **C**). However, it curbed haemolytic potential of *S. aureus* notably. Hemoglobin released as a result of haemolysis was quantified as OD _490_; 1% triton (OD _490_ = 1.2), and PBS (pH 7.4) were used as positive and negative control respectively; *p<0.05, ***p<0.001; TF: *Triphala* Formulation; PBS: Phosphate Buffer Saline.

**Figure 10.**
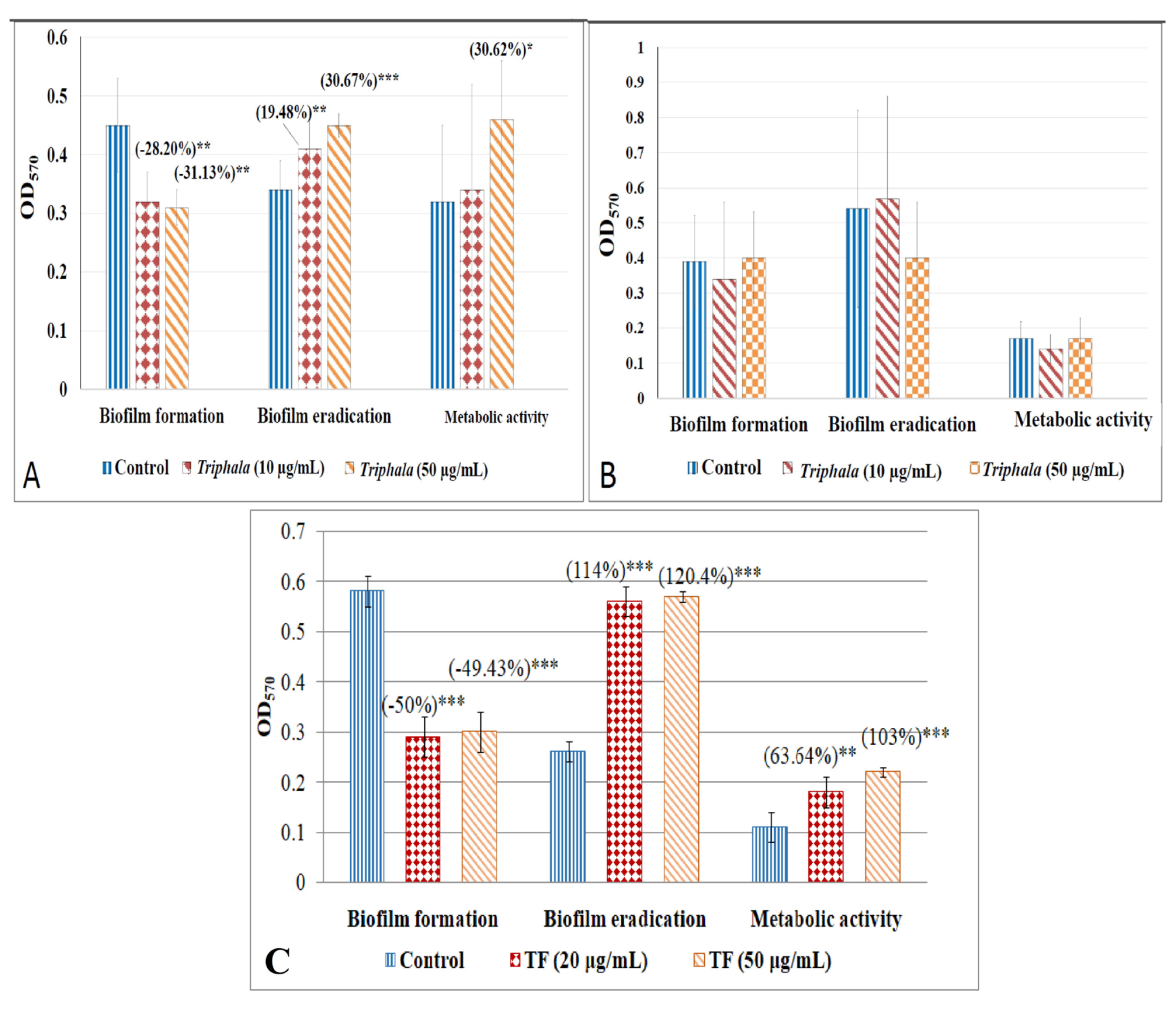
Effect of *Triphala* formulation on biofilm of test pathogens. Crystal violet assay was performed to quantify biofilm formation or eradication, wherein amount of this dye retained by the biofilm was read at 570 nm after extracting it in ethanol. MTT assay was performed to quantify biofilm viability (metabolic activity), wherein change in colour of the MTT dye owing to bacterial metabolism was read at 570 nm. Catechin (100 µg/ml) for all test bacteria, ampicillin (5 µg/ml) for *S. marcescens,* gentamicin (0.5 µg/ml) for *P. aeruginosa,* and vancomycin(0.1 µg/ml) for *S. aureus* were used as positive controls. ( **A**)Effect of TF on *S. marcescens* biofilm. Catechin inhibited *S. marcescens* biofilm formation by (29.80±9.38)%***, eradicated pre-formed biofilm by (49.60 ±12.00)%*, and reduced metabolic activity of biofilm by (86.20 ±0.88)% ***. Ampicillin inhibited all three by 29.25*** ±7.30, 54.23*** ±3.67, 86.11*** ±0.21 for *S. marcescens.* ( **B**) Effect of TF on *P. aeruginosa* biofilm. Catechin inhibited *P. aeruginosa* biofilm formation by (22.18±1.16)%***, eradicated pre-formed biofilm by (30.54 ±3.50)%**, and enhanced metabolic activity of biofilm by (32.27 ± 4.74 )%***. Gentamicin did not affect biofilm formation, eradicated pre-formed biofilm by (23.27±4.91)%***, reduced metabolic activity of biofilm by (123.99 ±26.81)% ***. ( **C**) Effect of TF on *S. aureus* biofilm. Catechin inhibited *S. aureus* biofilm formation by (26.24±7.35)%***, enhanced pre-formed biofilm by (22.54 ±10.90)%**, and enhanced metabolic activity of biofilm by (177.71 ± 16.49)% ***. Vancomycin inhibited biofilm formation by (41.53±5.49)% ***, eradicate pre-formed biofilm by (42.37±11.21)%***, enhanced metabolic activity of biofilm by (70.90 ±5.10)% ***. * *p*<0.05, ** *p*<0.01, ***p<0.001; TF: *Triphala* Formulation.

 We also tested the effect of TF on two important virulence traits of the bacterial pathogens i.e. haemolytic activity, and biofilm. Though TF could not curb haemolytic activity of any of the gram-negative bacteria, this activity of *S. aureus* was heavily inhibited under the influence of TF [ Figure 9; underlying data ( Patel *et al*., 2019c)]. While *P. aeruginosa* biofilm was not affected by TF, TF was able to reduce biofilm formation by *S. marcescens*, and *S. aureus*. When TF was applied on pre-formed bacterial biofilms, it seemed to enhance synthesis of the biofilm matrix material (quantified thorough crystal violet assay), and also the metabolic activity (measured in terms of organism's ability to reduce MTT) of the bacterial biofilm [ Figure 10; underlying data ( Patel *et al*., 2019c)]. It may be speculated that TF-treatment induces stress in the bacterial population residing in biofilm form, and this causes the bacteria to mount stress-response. Slow metabolism is a general characteristic of bacterial biofilms ( Singh *et al*., 2017), but TF seems to have forced the biofilms of two of our test bacteria to enhance the rate of their metabolic activity, as well as synthesis/secretion of biofilm matrix components (e.g. polysaccharides, proteins, and extracellular DNA). Enhanced production of exopolysaccharide and e-DNA is believed to occur in stressed bacterial populations ( Chang *et al*., 2007; Zatorska *et al*., 2018). Sub-inhibitory concentrations of beta-lactam antibiotics have been reported to induce extracellular DNA release and biofilm formation in some *S. aureus* strains ( Kaplan *et al*., 2012).

**Figure 11.**
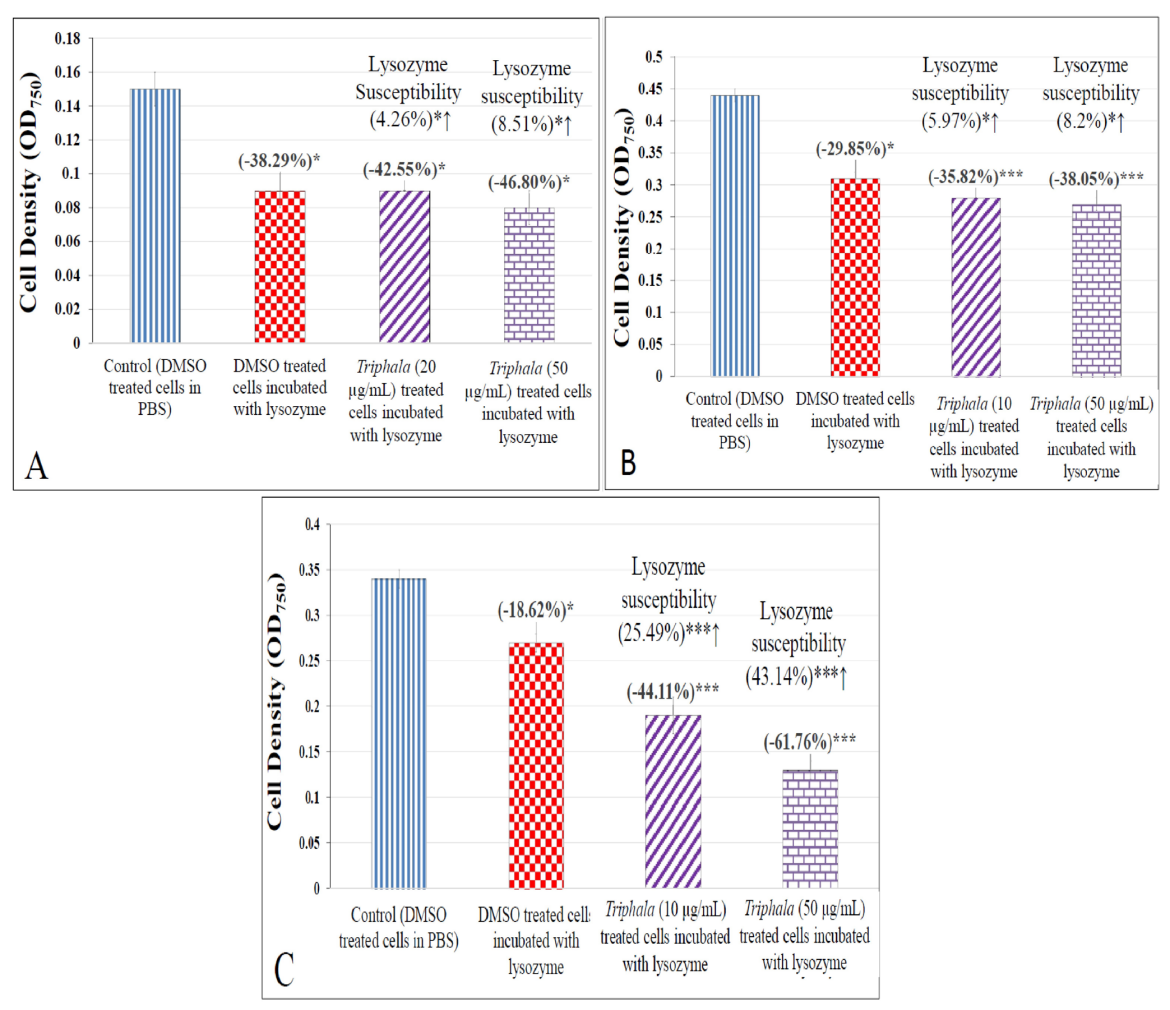
*Triphala* formulation increases susceptibility of test pathogens to lysozyme. ( **A**) *S. aureus*; ( **B**) *S. marcescens*; ( **C**) *P. aeruginosa* *p≤0.05 ***p≤0.001.

 During the host-pathogen interaction, host defense mechanisms play a determinant role in deciding the outcome of this interaction. Since lysozyme is an important component of the innate defense machinery of human immune system against invading microbes ( Herbert *et al*., 2007), we also studied whether TF can have any effect on susceptibility of the test pathogens to lysozyme. TF-treated cells of *S. marcescens* and *S. aureus* were found to suffer marginal (albeit statistically significant; p≤0.05) increase in their susceptibility to lysis by lysozyme. *P. aeruginosa's* lysozyme-susceptibility was found to increase heavily (by ~25-43%) upon TF-pretreatment [ Figure 11; underlying data ( Patel *et al*., 2019c)].

 Most conventional antibiotics suffer from an inherent limitation of not being selectively inhibitory to pathogenic bacteria, and they simultaneously inhibit resident bacterial members of the human microbiome; which may lead to gut dysbiosis ( Wipperman *et al*., 2017). Thus an ideal anti-pathogenic formulation should exert anti-pathogenic effects without inhibiting indigenous members of human microbiome. We tested TF's effect on three such bacteria ( *Enterococcus faecium*, *Bifidobacterium bifidum*, and *Lactobacillus plantarum*) which are part of human microbiome, and also used as probiotic strains. Though TF did not exert any prebiotic potential by promoting growth of the probiotic bacteria, it also had no negative effect on them [Extended data: Figure S1 ( Patel *et al*., 2019c)].

## Conclusion

This study has found the classical TF to possess significant anti-infective potential against gram-positive and gram-negative pathogenic bacteria. It was also found to be efficacious as a post-infection therapy as well as a prophylactic measure against bacterial infection. TF can be said to possess a broad-spectrum of anti-pathogenic activity, which seems to partly arise from its ability to interfere with bacterial quorum-sensing. Its prophylactic efficacy indicates that it is not only exerting inhibitory effect on the susceptible bacteria, but also beneficial effect on the host worm, and thus can be described as a combination of immunomodulatory and anti-pathogenic activities in one formulation. Exerting such combined efficacy without displaying any negative effect on beneficial members of human microbiome are key attributes for 21 ^st ^century antimicrobials ( Laxminarayan *et al*., 2013). Further investigation for elucidating the molecular mechanisms associated with the biological effects of TF are warranted, with special emphasis on its role in combating AMR. Such traditional medicine polyherbal formulations need not necessarily be thought of as replacement of conventional antibiotic treatments, but more realistically as adjunctive therapies boosting our efforts to tackle AMR effectively.

## Data availability

### Underlying data

Figshare: Anti-pathogenic potential of a classical ayurvedic formulation- Triphala. https://doi.org/10.6084/m9.figshare.8052143.v2 ( Patel *et al*., 2019c)

- Raw data_Figures 1-11_S1.rar

### Extended data

Figshare: Anti-pathogenic potential of a classical ayurvedic formulation- Triphala. https://doi.org/10.6084/m9.figshare.8052143.v2 ( Patel *et al*., 2019c)

This project contains the following extended data:

- Video (a).avi (Video of *C. elegans* challenged with *S. marcescens*)
- Video (b).avi (Video of *C. elegans* exposed to TF-treated *S. marcescens*)
- Figure S1.jpg (effect of TF treatment on probiotic bacterial strains)

Data are available under the terms of the Creative Commons Zero "No rights reserved" data waiver (CC0 1.0 Public domain dedication).
