# Supplementary material for: Anti-pathogenic potential of a classical ayurvedic  Triphala formulation
Source: F1000Res. 2020 Oct 1;8:1126. Originally published 2019 Jul 18. [Version 2] doi: 10.12688/f1000research.19787.2 (PMC7549186; doi:10.12688/f1000research.19787.2)
Supplement: Peer review history

# Peer review: Reviewer response for version 2

**DOI:** 10.5256/f1000research.29732.r72330

**Published:** 2020-10-09

**Authors:** Shibabrata Pattanayak

**Affiliations:** 1 ARD (Vet. Research & Investigation), Government of West Bengal, Kolkata, West Bengal, India

The article may be accepted and indexed at the present form.

Reviewer Expertise:

Ethno- Pharmacology, Immunology, Bacteriology, Virology, Biochemistry

I confirm that I have read this submission and believe that I have an appropriate level of expertise to confirm that it is of an acceptable scientific standard.

# Peer review: Reviewer response for version 2

**DOI:** 10.5256/f1000research.29732.r72329

**Published:** 2020-10-02

**Authors:** Felipe Alves de Almeida

**Affiliations:** 1 Department of Nutrition, Federal University of Juiz de Fora (UFJF), Governador Valadares, Brazil

I have no further comments to make.

Reviewer Expertise:

Microbiology.

I confirm that I have read this submission and believe that I have an appropriate level of expertise to confirm that it is of an acceptable scientific standard.

# Peer review: Reviewer response for version 1

**DOI:** 10.5256/f1000research.21707.r56187

**Published:** 2020-09-02

**Authors:** Felipe Alves de Almeida

**Affiliations:** 1 Department of Nutrition, Federal University of Juiz de Fora (UFJF), Governador Valadares, Brazil

The article is interesting, but some adjustments are needed. Initially, the quality of the figures should be improved. One point that concerns me is the interpretations of the results of the *in vitro *experiments, because when the concentration of TF influences the growth of bacteria, nothing can be said about its anti-quorum sensing effect. The other comments are marked in a document in PDF format which can be found here.

Is the work clearly and accurately presented and does it cite the current literature?

Yes

If applicable, is the statistical analysis and its interpretation appropriate?

Yes

Are all the source data underlying the results available to ensure full reproducibility?

Yes

Is the study design appropriate and is the work technically sound?

Yes

Are the conclusions drawn adequately supported by the results?

Partly

Are sufficient details of methods and analysis provided to allow replication by others?

Yes

Reviewer Expertise:

Microbiology.

I confirm that I have read this submission and believe that I have an appropriate level of expertise to confirm that it is of an acceptable scientific standard, however I have significant reservations, as outlined above.

# Response

**Published:** 2020-09-18

**Authors:** Vijay Kothari

**Affiliations:** Nirma university, India

**Response to Referees**

 We thank both the referees for devoting their valued time in reviewing our manuscript. Our comment-wise response to their comments is as under:

**Response to general comments from Dr. Felipe Alves de Almeida:**

1. Quality of the figures: We have set the resolution of all figures at 1000 dpi (which also satisfies journal’s criteria of minimum 600 dpi), and are unable to improve them further.
2. The referee has pointed to a very logical issue with respect to interpretation of the *in vitro* results that if the test formulation affects bacterial growth, then how can we judge its effect on pigment production as quorum-modulatory effect?

To safeguard against this, we have already plotted ‘Pigment Unit’, and not the absolute pigment OD. ‘Pigment Unit’ are OD of pigment divided by cell density. Since this is a quantification of pigment produced per unit of growth, it nullifies any variation in pigment production due to changes in cell density in experimental vs. control tubes. Calculation of these ‘Pigment Units’ for individual pigments has already been mentioned in the legend of Figure-8. To further clarify this point, we have added this sentence to the figure-8 legend: “Pigment Units were calculated to nullify the effect of any change in cell density on pigment production”.

 On the same line, for other *in vitro* assays too (e.g. lysozyme susceptibility, haemolysis assay, etc.), before performing the assay we had equalized cell density of the *control *and *experimental *cultures, to ensure that TF’s effect (if any) on bacterial growth does not interfere with interpretation of our results. This OD equalization step has already been mentioned in the ‘Methods’ section.

**Response to comments from Dr. Felipe Alves de Almeida marked within the annotated manuscript:**

1. The referee has asked for why have we chosen 750 nm for quantifying cell density. Please note that the bacteria being studied here are pigmented ones, and their pigments can interfere in measuring cell density within the visible range of spectrum (400-700 nm). In our earlier study [Joshi et al. (2016); DOI: 10.2174/1872208310666160414102848; which we have already cited in the current paper], we have shown 764 nm as the more appropriate one for quantifying cell density of pigmented bacteria, as compared to the conventionally used wavelengths e.g. 600 nm. Since the wavelength nearest to 764 nm available in the instrument used by us was 750 nm, we used that. We had earlier also confirmed that OD of the bacterial suspension used by us do not statistically differ at 764 nm vs. 750 nm. Appropriateness of 764 nm for studying *C. violaceum* has also been reported by Gallardo et al. AMB Express 2014, 4:4 [http://www.amb-express.com/content/4/1/4].
2. In the ‘Results’ section under ‘in vivo assays’, the referee has raised a question that why different concentrations of TF used for pathogens. In response to that we would like to state that all pathogens were challenged with identical concentrations of TF in the range of 0.5-100 microgram/mL. However in figures corresponding to these results, we have showed only non-overlapping lines. Data regarding other concentrations has been mentioned in the figure legends. If we include lines pertaining to all concentrations in the graph, then it becomes very fuzzy and difficult to understand.
3. Though referee’s suggestion of merging Figure-2 with Figure-1 is fully logical, we are unable to implement it, as that will cause multiple lines in Figure-1 to overlap making it fuzzy and difficult to understand. For the same reason, we were not able to put lines for all TF concentrations in the graphs of Figure-1, and had to include that data in figure legend.
4. Referee has suggested for including the details of Post-Extract Effect (PEE) in ‘Method’ section. However that was not done as the method is not different than that described for ‘anti-infective assay’ except that in the PEE assay the host worm was challenged with daughter cells of the TF-treated parent cells; and this small piece of  information has already been mentioned in the ‘Results’ section while first mentioning PEE.
5. The referee has asked for data of PEE against *C. violaceum*. However PEE assay was done only with the pathogenic bacteria which are considered as  serious threats, i.e. whose pathogenic/ antibiotic-resistant strains are listed as priority pathogens by CDC/ WHO.
6. The referee has raised a query about the difference in survival % of unchallenged/ unfed worms between figure 5 and figure 6. It should be noted that prior to the prophylactic assay, whose results are depicted in Figure-5, worms were kept unfed for 5 days; whereas prior to longevity assay (results depicted in Figure-6) the worms were kept unfed for 3 days. Hence this additional 2-dyas of non-availability of food in the prophylactic assay explains lower survival of worms in ‘control’ wells in Figure-5.
7. As suggested by the referee, we have replaced ‘Triphala formulation’ with ‘TF’ at multiple places. Similarly at many places, he has suggested removal of parentheses, that has also been implemented.
8. Figure-8 has been revised as per his suggestion.
9. Figure-10: Referee has raised query about accuracy of the % values. We thank him for his fine observation, as investigating this has made us locate errors in some % values as well as bar heights. All those errors have been corrected, and we are submitting revised figure.
10. All other minor corrections/ modifications marked by the referee in the PDF file have also been either implemented, OR we have provided appropriate response/ explanation by inserting ‘comments’ in the word file of revised version.

 

 We hope that the revised version along with clarifications provided by us will be able to earn referee’s and editor’s approval.

# Peer review: Reviewer response for version 1

**DOI:** 10.5256/f1000research.21707.r51849

**Published:** 2019-08-15

**Authors:** Shibabrata Pattanayak

**Affiliations:** 1 ARD (Vet. Research & Investigation), Government of West Bengal, Kolkata, West Bengal, India

**Following remarks are to be considered.**

**A.Test formulation**

*Triphala *formulation (TF) (Emami Ltd; batch no. EM0029; Proportion of 3 constituent plant species: 1:1:1) was purchased from a local market. For assay purpose, 150 mg of this formulation was suspended in 5 ml of DMSO (Merck, Mumbai), followed by vortexing for 15 min. Then it was centrifuged at 8,000 rpm for 30 min at ambient temperature, and resulting supernatant was collected in a sterile glass vial (15 ml; Borosil) and stored under refrigeration till further use. Remaining pellet was subjected to drying in an oven at 70-80°C until the solvent was completely evaporated, followed by weighing of the dried plant material. Subtracting the latter from the initial weight of 150 mg, the concentration of test formulation in supernatant was calculated to be 22.94 mg/ml. This way the whole formulation was found to contain 70% DMSO soluble fraction, which was used for our experiments.

**Remark**

 DMSO is having some effect on at least some microorganisms. What concentration of DMSO was used? Please add report / reference of non – germicidal effect of DMSO at the concentration used in your experiment.

 You may consult the following articles:

1. Kirkwood ZI, Millar BC, Downey DG, Moore JE. Antimicrobial effect of dimethyl sulfoxide and *N, N- *Dimethylformamide on *Mycobacterium abscessus*: Implications for antimicrobial susceptibility testing. Int J Mycobacteriol 2018;7:134-136. ^1^
2. Mi H, Wang D, Xue Y, Zhang Z, Niu J, Hong Y, Drlica K, Zhao X. 2016. Dimethyl sulfoxide protects *Escherichia coli *from rapid antimicrobial-mediated killing. Antimicrob Agents Chemother 60:5054 –5058. doi:10.1128/AAC.03003-15. ^2^
3. Ashraf S. Hassan (2014) The Antibacterial Activity of Dimethyl Sulfoxide (DMSO) with and without of Some Ligand Complexes of the Transitional Metal Ions of Ethyl Coumarin against Bacteria Isolate from Burn and Wound Infection. Journal of Natural Sciences Research. Vol.4, No.19, 106-111. ^3^
4. Howard C. Ansel, William P. Norred, Ivan L. Roth (1969) Antimicrobial activity of dimethyl sulfoxide against *Escherichia coli, Pseudomonas aeruginosa*, and *Bacillus megaterium.* J  Pharmaceutical sciences 58(7): 836-839. ^4^

**B. Some portions are appeared unclear. Please modify the sentences:**


**  **

1. **Page 2, Column 2 Paragraph 4.**
  Prophylactic assay (Patel *et al*., 2018b): …..These worms were then fed with TF by mixing required concentration of this formulation (100 μL) with M9 medium (800 μL) and placed in a 24-well plate (non-treated polystyrene plates….
**Remark:**
**Is it the DMSO –TRIFALA mixed supernatant?**
2. **Page 4 Column 1 Paragraph 4.**
  …..at par with that of control (DMSO treated parent culture). –
**Remark:**
**Point is not clear.** 

**Overall Comment:**

**The research work is very good. The writing style is also good. After the mentioned modifications, the article may be accepted.**

Is the work clearly and accurately presented and does it cite the current literature?

Partly

If applicable, is the statistical analysis and its interpretation appropriate?

I cannot comment. A qualified statistician is required.

Are all the source data underlying the results available to ensure full reproducibility?

Yes

Is the study design appropriate and is the work technically sound?

Partly

Are the conclusions drawn adequately supported by the results?

Yes

Are sufficient details of methods and analysis provided to allow replication by others?

Yes

Reviewer Expertise:

Ethno- Pharmacology, Immunology, Bacteriology, Virology, Biochemistry

I confirm that I have read this submission and believe that I have an appropriate level of expertise to confirm that it is of an acceptable scientific standard, however I have significant reservations, as outlined above.

## References

[1] : Antimicrobial effect of dimethyl sulfoxide and N, N-Dimethylformamide on Mycobacterium abscessus: Implications for antimicrobial susceptibility testing. *Int J Mycobacteriol*.7(2) : 10.4103/ijmy.ijmy_35_18 134-136 10.4103/ijmy.ijmy_35_18 29900888

[2] : Dimethyl Sulfoxide Protects Escherichia coli from Rapid Antimicrobial-Mediated Killing. *Antimicrob Agents Chemother*.60(8) : 10.1128/AAC.03003-15 5054-8 10.1128/AAC.03003-15 27246776PMC4958201

[3] : The Antibacterial Activity of Dimethyl Sulfoxide (DMSO) with and without of Some Ligand Complexes of the Transitional Metal Ions of Ethyl Coumarin against Bacteria Isolate from Burn and Wound Infection. *Journal of Natural Sciences Research*.2014;4(19) :106-111

[4] : Antimicrobial activity of dimethyl sulfoxide against Escherichia coli, Pseudomonas aeruginosa, and Bacillus megaterium. *J Pharm Sci*.1969;58(7) : 10.1002/jps.2600580708 836-9 10.1002/jps.2600580708 4980332

# Response

**Published:** 2020-09-18

**Authors:** Vijay Kothari

**Affiliations:** Nirma university, India

We thank both the referees for devoting their valued time in reviewing our manuscript. Our comment-wise response to their comments is as under:

**Response to comments from Dr. S Pattanayak:**

1. DMSO was used in our experiments at 0.5%v/v. This information along with the fact that this much DMSO did not affect growth / pigment production / virulence of the bacterial strains employed in this study has already been there in legends of all the figures. For example, absence of *in vitro* effect of DMSO on growth and pigment production has been reported in legend of Figure-8. Legend of Figure-1 includes the statement that DMSO (0.5%v/v) affected neither bacterial virulence, nor did it exert any toxicity toward the host worms.

Referee has very logically raised this concern, as DMSO at higher concentrations have been reported to affect bacterial growth by multiple researchers including our group [doi: 10.1016/S1995-7645(14)60233-9;] and those cited in the referee report. However, since we have already included the ‘vehicle control’ in all our experiments; we did not cite any separate references for that. DMSO at the concentration (0.5%v/v) used in this study has earlier been reported by us to exert no effect on bacterial growth and pigment production: https://shodhganga.inflibnet.ac.in/bitstream/10603/245830/14/17_annexure.pdf

2. Yes, it is *Triphala *dissolved in DMSO, which was fed to the worm population during prophylactic assay. The sentence “These worms were then……………….” has been modified.

3. For the sake of brevity, we have modified the sentence: “………..at par with………….”. Here what we could demonstrate is that effect of TF remains even on the ‘daughter cells’ of the TF-exposed ‘parent bacterial cells’. It should be noted that these daughter cells themselves were never directly exposed to the test formulation, and still their virulence is attenuated owing to their parent population’s exposure to TF. To make the concept of ‘post-extract effect’ (PEE) clear to the readers, we have already provided a link [https://doi.org/10.32388/359873] in the same paragraph, which takes the reader to the definition of PEE. 

 We hope that the revised version along with clarifications provided by us will be able to earn referee’s and editor’s approval.
